# Predicting and Analyzing Protein Phosphorylation Sites in Plants Using Musite

**DOI:** 10.3389/fpls.2012.00186

**Published:** 2012-08-21

**Authors:** Qiuming Yao, Jianjiong Gao, Curtis Bollinger, Jay J. Thelen, Dong Xu

**Affiliations:** ^1^Department of Computer Science, University of MissouriColumbia, MO, USA; ^2^Bond Life Science Center, University of MissouriColumbia, MO, USA; ^3^Computational Biology Center, Memorial Sloan-Kettering Cancer CenterNew York, NY, USA; ^4^Department of Biochemistry, University of MissouriColumbia, MO, USA

**Keywords:** Musite, phosphorylation, machine-learning, orthologous, protein disorder, plant evolution

## Abstract

Although protein phosphorylation sites can be reliably identified with high-resolution mass spectrometry, the experimental approach is time-consuming and resource-dependent. Furthermore, it is unlikely that an experimental approach could catalog an entire phosphoproteome. Computational prediction of phosphorylation sites provides an efficient and flexible way to reveal potential phosphorylation sites and provide hypotheses in experimental design. Musite is a tool that we previously developed to predict phosphorylation sites based solely on protein sequence. However, it was not comprehensively applied to plants. In this study, the phosphorylation data from *Arabidopsis thaliana*, *B. napus*, *G. max*, *M. truncatula*, *O. sativa*, and *Z. mays* were collected for cross-species testing and the overall plant-specific prediction as well. The results show that the model for *A. thaliana* can be extended to other organisms, and the overall plant model from Musite outperforms the current plant-specific prediction tools, Plantphos, and PhosphAt, in prediction accuracy. Furthermore, a comparative study of predicted phosphorylation sites across orthologs among different plants was conducted to reveal potential evolutionary features. A bipolar distribution of isolated, non-conserved phosphorylation sites, and highly conserved ones in terms of the amino acid type was observed. It also shows that predicted phosphorylation sites conserved within orthologs do not necessarily share more sequence similarity in the flanking regions than the background, but they often inherit protein disorder, a property that does not necessitate high sequence conservation. Our analysis also suggests that the phosphorylation frequencies among serine, threonine, and tyrosine correlate with their relative proportion in disordered regions. Musite can be used as a web server (http://musite.net) or downloaded as an open-source standalone tool (http://musite.sourceforge.net/).

## Introduction

Protein phosphorylation plays important roles in numerous cellular processes in plants. Although mass spectrometry based studies have provided high-throughput phosphorylation data, it is still time-consuming and expensive to identify phosphorylation sites experimentally. Computational prediction of phosphorylation sites directly from protein sequences provides an alternative approach. A number of software tools have been developed under this provenance, such as NetPhos (Blom et al., 1999), Scan-x (Schwartz et al., 2009), and DISPHOS (Iakoucheva et al., 2004). We recently developed Musite (Gao et al., 2010), which incorporates feature selection and machine-learning processes as well as other useful tools into one open-source frame work. It is computationally efficient, offers a statistical assessment of data quality, and can handle proteome-wide prediction.

However, when Musite was initially developed, phosphorylation site data from plants were sparse and insufficient to train and test beyond model plants. The only large dataset was *Arabidopsis* (*Arabidopsis thaliana*) data from PhosPhAt with 3,159 phospho-serine sites and 504 phospho-threonine sites (Heazlewood et al., 2008). Hence, Musite did not have a general plant phosphorylation predictor except for *Arabidopsis*. This was also the case for other tools that support plant phosphorylation site prediction such as DISPHOS (Iakoucheva et al., 2004). Since Musite was first published (Gao et al., 2010), more experimental plant phosphorylation sites have become available. The Plant Protein Phosphorylation Database (P^3^DB; Gao et al., 2009) now contains 32,963 non-redundant sites collated from 23 experimental studies from six plant species (*A. thaliana*, *B. napus*, *G. max*, *M. truncatula*, *O. sativa*, and *Z. mays*), providing a good opportunity to train and test for a general plant phosphorylation site predictor.

As the coverage of experimental phosphorylation sites in plants increases, there have been a few studies on conservation patterns of phosphorylation sites among different plants. It was observed that some phosphorylation sites and peptides are conserved within the gene families across different species (Maathuis, 2008). A few comparative phosphoproteomics studies explored and revealed evolutionary patterns in terms of conserved, functional phosphorylated sites (Boekhorst et al., 2008; Nakagami et al., 2010; Meyer et al., 2012). To complement these studies, it is interesting to explore the evolutionary patterns in the predicted phosphoproteome as well. Although predicted phosphorylation sites are putative, the statistical patterns may be similar between the predicted and *bona fide* phosphorylation sites. An advantage of using predicted phosphorylation sites is that coverage is higher and less biased (in terms of protein abundance, etc.) than experimentally identified phosphorylation sites.

In this paper, a Musite prediction model based on *Arabidopsis* data only was trained and cross-organism testing was then performed on other plants. A phosphorylation prediction model for plants was also trained from combined plant phosphorylation data. Predicted phosphorylation sites in orthologous groups off our green plants were compared to reveal potential evolutionary trends. In addition, analysis on distribution of protein disorder in predicted phosphorylation peptides was performed and provides some hypotheses of the phosphorylation tendency and evolutionary tends.

## Materials and Methods

### Datasets

The phosphorylation sites being analyzed were from six organisms, i.e., *A. thaliana* (Nuhse et al., 2004, 2007; Wolschin and Weckwerth, 2005; de la Fuente van Bentem et al., 2006, 2008; Benschop et al., 2007; Sugiyama et al., 2008; Whiteman et al., 2008; Hsu et al., 2009; Ito et al., 2009; Jones et al., 2009; Li et al., 2009; Reiland et al., 2009; Wang et al., 2009; Chen et al., 2010; Kline et al., 2010; Nakagami et al., 2010; Engelsberger and Schulze, 2012; Meyer et al., 2012), *B. napus* (Meyer et al., 2012), *G. max* (Meyer et al., 2012), *M. truncatula* (Grimsrud et al., 2010), *O. sativa* (Nakagami et al., 2010), and *Z. mays* (Bi et al., 2011). These phosphorylation sites were downloaded from P^3^DB version 2.0 (Gao et al., 2009; Yao et al., submitted) and phosphorylation annotations in UniProt release 2012_02 (Farriol-Mathis et al., 2004). For each organism, the proteins collected from both sources were merged into a single dataset. More specifically, if a phosphorylation site was observed in any one source, it was used as positive data. The non-phosphorylated proteins of the correspondent organism without any phosphorylation annotation were used as negative data.

The sequence-wide redundancy was removed in order to avoid potential bias in the machine-learning training process. CD-HIT (Li and Godzik, 2006) was used in this process and proteins with more than 50% of sequence identity were removed. For *A. thaliana*, the non-phosphorylated proteins dominate the whole dataset. When training, we sampled the negative data to create a balanced dataset at the protein level. While the balance at the protein level does not mean the same numbers of phosphorylated sites and non-phosphorylated sites, the balance at the phosphorylated site level was handled by the bootstrapping procedure described below. The distribution of the datasets is shown in Table 1.

**Table 1:** **Training and testing datasets**.

	Organism	Phosphoproteins	Phosphoserines	Phosphothreonines	Phosphotyrosine
Train	*A. thaliana*	2,000/4,000	5,372/195,854	1,441/103,593	420/54,944
Test	*A. thaliana*	484/968	1,433/46,758	361/24,353	107/13,082
	*B. napus*	184/285	285/6,426	133/4,133	58/2,056
	*G. max*	949/1,201	1,381/49,288	235/25,680	67/13,762
	*M. truncatula*	701/733	2,003/42,077	378/20,645	79/9,912
	*O. sativa*	2,604/2,685	5,250/147,508	862/78,928	244/40,432
	*Z. mays*	68/528	78/12,822	11/8,800	0/5,037

*“/” Separates the number of positive sites and the number of total sites*.

### Machine-learning framework

The phosphorylation prediction is formulated as a binary classification problem, which can be modeled and solved by a machine-learning framework (Gao et al., 2010). *K* Nearest Neighbor (*K*NN) scores, disorder scores, and amino acid frequencies were used as the features for the training. The serine-, threonine-, or tyrosine-centered flanking sequences were used as peptide samples to extract the features. Practically, the length of the flanking sequences is not fixed. Multiple sizes of the peptides represent different scales of the local properties.

*K* nearest neighbor score is the ratio between the numbers of positive and negative sites among the pre-defined number of neighbors of a given peptide. The neighborhood of a peptide is defined as a certain percentage of the training dataset ranked by the pair-wise similarity between the target peptide and the peptides in the dataset. In this study, the sequence similarity calculation was based on the BLOSUM62 matrix; the neighborhood was set to 0.25, 0.5, 1, 2, and 4% of the training dataset, respectively; and the flanking sequence size was set to 13 amino acids (±6 at each side).

Disorder score is a feature to measure the stability of the local structure. It was calculated by VSL2B (Obradovic et al., 2005), a widely used predictor of protein disorder from sequence only. The disorder score of a given peptide was calculated as the average disorder score over all its amino acids. In this study, the sequence length of the disorder calculation was set as 1, 5, and 13 amino acids.

Amino acid frequency reflects the amino acid preference in phosphorylated peptides (Iakoucheva et al., 2004). It was represented as a vector of length of 20, which contained the normalized counts for every type of amino acid. The length of the peptide for calculating amino acid frequency was set to 13.

Bootstrapping was applied to solve the unbalanced problem of the positive and negative sites. When training, the same number of negative sites as positive sites was randomly sampled and formed a balanced set for training. Then, a support vector machine (SVM; Joachims, 2002) was used to train multiple prediction models on the sampled datasets. When predicting, the final prediction score was aggregated by averaging the outputs of all the SVM classifiers.

### Cross-organisms testing

The SVM model was trained on *A. thaliana*. The testing was first performed on a new dataset of *A. thaliana* to evaluate if the model performance was consistent or not. The main testing cases were on other organisms, as described above. The testing results were presented as receiver operating characteristic (ROC) curves, with “1-specificity” (i.e., false positive rate) vs. “sensitivity” (i.e., true positive rate) on horizontal and vertical axes, respectively. Specificity and sensitivity are defined as follows:

(1) $$\text{specificity}=\frac{\text{TN}}{\text{TN}+\text{FP}},\;\text{sensitivity}=\frac{\text{TP}}{\text{TP}+\text{FN}}$$

where TN represents true negative, FP false positive, TP true positive, and FN false negative.

### Orthologous group analysis

The orthologous groups among four organisms (*A. thaliana*, *O. sativa*, *R. communis*, and *P. patens*) were collected from OrthoMCL orthologous dataset (Li et al., 2003; Chen et al., 2007). The data for other plants were unavailable in OrthoMCL so that they were not included in this study. The protein sequences needed for the orthologous groups were downloaded from Phytozome (Goodstein et al., 2012). In each group, the four sequences of an orthologous group were aligned globally by MUSCLE (Edgar, 2004) and their phosphorylation sites were predicted by Musite. Then the peptides centered by serine, threonine, or tyrosine were extracted and classified into the S/T class and the Y class according to the center amino acid. By analyzing the aligned sequences within each orthologous group and checking their predicted phosphorylation states, we studied potential phosphorylation conservation patterns. If at least one site in the center position of an orthologous group was predicted as a phosphorylation site, we included the orthologous group to study the distribution of the phosphorylated centers in terms of their phosphorylation states, which could be 1, 2, 3, or 4 phosphorylation sites. This can be modeled by a binomial distribution and therefore the chi-square test was used to test if the expected polynomial distribution is the same as the observed distribution, i.e.,

(2) $$\chi^{2}=\sum_{i=1}^{4}\frac{{\left(\text{Observe}{\text{d}}_{i}-\text{Expecte}{\text{d}}_{i}\right)}^{2}}{\text{Expecte}{\text{d}}_{i}}$$

with three degrees of freedom.

## Results

**Figure 1:**
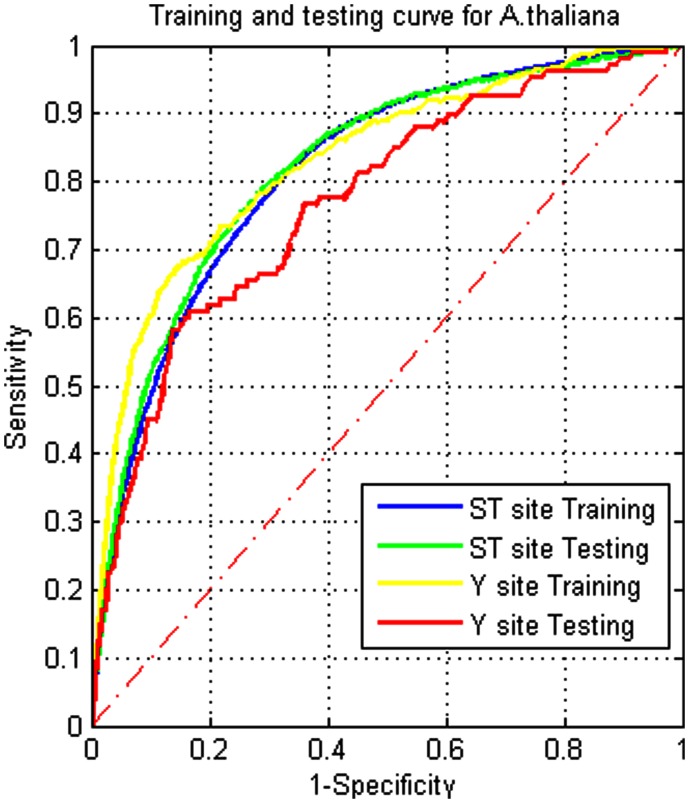
**Receiver operating characteristic curves for training and testing on *Arabidopsis***.

**Figure 2:**
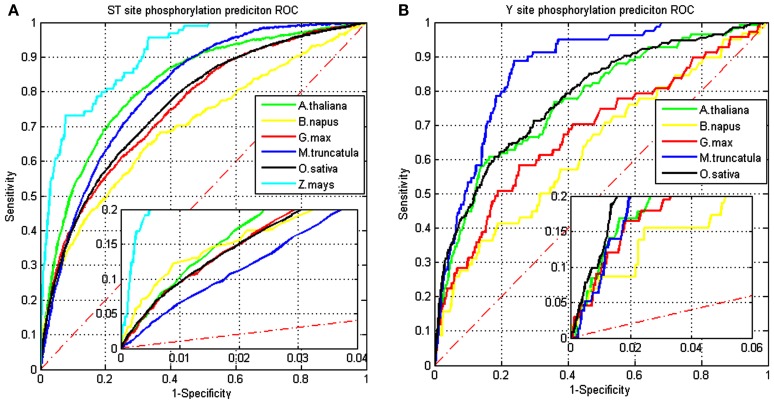
**Receiver operating characteristic curves for testing on different organisms using the trained model from *A. thaliana*: (A) curves for S/T site predictions and (B) curves for Y site predictions**.

**Figure 3:**
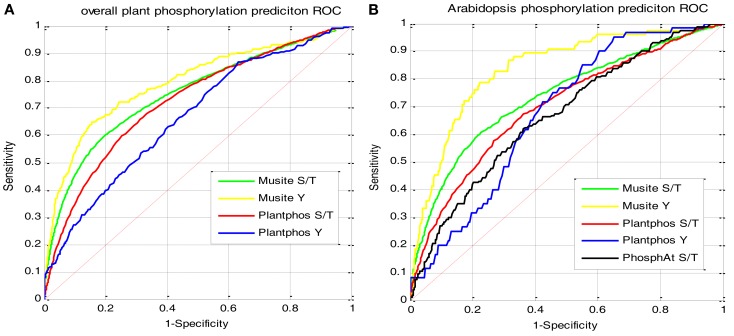
**Comparison of ROC curves on different plant-specific tools**. For Musite, the training set is on 3/4 of the total phosphorylation data from six organisms. **(A)** Testing result on the rest of 1/4 phosphorylation data, in comparison with Plantphos; **(B)** testing result on *Arabidopsis* only from the dataset in **(A)** in order to compare with PhosphAt.

For the trained model from *A. thaliana*, the ROC curves for the prediction results were plotted in Figures 1–3. Figure 1 indicates that the performance between training and testing is similar, especially for the S/T class, i.e., over-training is not a concern. *Z. mays* does not have an ROC curve for Y sites since no experimental data for tyrosine phosphorylation are currently available.

The same model was applied to the other five organisms (*B. napus*, *G. max*, *M. truncatula*, *O. sativa*, and *Z. mays*) for S/T and Y phosphorylation site prediction. Figure 2 shows ROC curves of the results for these five plant organisms. For the S/T site prediction, the model performs roughly equally well on *A. thaliana*, *G. max*, *M. truncatula*, and *O. sativa*. *Z. mays* has the best performance and *B. napus* has the worst performance. For the Y site prediction, the model performs similarly between *O. sativa* and *A. thaliana*. Performance of *M. truncatula* is much better than those of *G. max* and *B. napus*.

An overall model of all the above plants species combined was also trained. For the sake of comparison, a randomly selected 3/4 training and 1/4 testing strategy was used, and the performance is shown in Figure 3. Figure 3A shows the comparison with Plantphos (Lee et al., 2011) on the 1/4 testing data. The Musite model outperformed Plantphos on both S/T sites and Y sites. To compare with PhosphAt (Heazlewood et al., 2008), an *Arabidopsis*-specific predictor, we used the *Arabidopsis* data from our 1/4 testing set that have the predictions from PhosphAt. Because the tyrosine phosphorylation predictions from PhosphAt were very sparse, we could not develop an ROC curve for tyrosine phosphorylation prediction. Figure 3B shows that Musite prediction outperformed PhosphAt. It is worth mentioning that in both testing cases, Musite did not include any testing data in its training set, while Plantphos and PhosphAt may have had some test data in their training, which could give them an advantage. Nevertheless, Musite’s improvement over these tools is significant.

**Figure 4:**
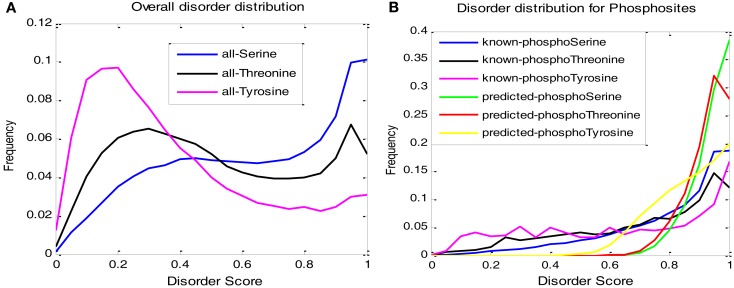
**Distribution of disorder scores: (A) Distribution for all S, T, and Y sites; (B) distribution of phosphosites for both known and predicted cases**.

Figure 4 shows the disorder score distributions. Figure 4A gives the disorder distribution on all the sites that we collected (phospho- and non-phospho-) showing that tyrosine tends to be in the ordered region while serine prefers disorder more than the other two amino acid types. Figure 4B provides the distributions of known phosphosites and predicted sites. The relative trends of serine, threonine, and tyrosine were similar between known and predicted sites, although the predicted sites are more enriched in high disorder regions.

Analysis of orthologous groups can help reveal conservation patterns of S/T and Y phosphorylation sites. The analysis was conducted among the phosphorylated groups (at least one center of the four in an orthologous group is phosphorylated). The motivation of this analysis was to determine the transfer probability of phosphorylation from one organism (one center) to other organisms (the other three centers in the orthologous group). The total number of extracted orthologous groups containing S/T or Y at the centers was 225,265. Two classes with pure S/T center or Y center were of interest. In the S/T centered class, there were 631 groups having at least one center predicted to be phosphorylated, and there were 200 in the corresponding Y centered class (see Table 2).

**Table 2:** **Statistics of S/T and Y centered classes**.

	1	2	3	4	Total
S/T (no. of orthologous groups)	291	156	82	102	631
S/T (%)	0.461	0.247	0.130	0.162	1
Expected no. of groups	189	280	139	23	631
Conditional probability		0.331	0.421	0.554	
Y (no. of orthologous groups)	110	50	25	15	200
Y (%)	0.550	0.250	0.125	0.075	1
Expected no. of groups	87	83	27	3	200
Conditional probability		0.242	0.688	0.375	

Given one phosphorylated center, if the other three centers are phosphorylated randomly, the distribution should yield a binomial distribution. As an example, under this assumption, for the S/T class, the expected probability of having a second phosphorylated center among the three remaining centers is P(S/T) = (1 × 156 + 2 × 82 + 3 × 102)/(631 × 3) = 0.331. For the Y class, the expected probability for a second phosphorylated center is P(Y) = (1 × 50 + 2 × 25 + 3 × 15)/(200 × 3) = 0.242. The expected number of orthologous groups in the same class (S/T or Y) having a second phosphorylated center is the total number of sites multiplied by the corresponding probability. Using a similar method, the expected numbers of orthologous groups containing four predicted phosphorylation sites were 23 and 3, respectively for S/T and Y classes vs. 102 and 15 observed orthologous groups of such cases, respectively. This indicates that phosphorylation is not a random process in evolution. The chi-square test was then performed between the observation and expectation based on Eq. 2. The statistic of chi-square test was 0.641 and 0.337 for S/T and Y classes, and the *p*-value for each was 0.113 and 0.047, respectively. This result reveals 89 and 95% of confidence for S/T and Y sites, respectively, indicating that phosphorylation is not a random factor in evolution.

Table 2 also lists the conditional probability of having an additional phosphorylation site in an orthologous group. Since the study is on the orthologous phosphorylation groups, we always consider that one of the four centers is already phosphorylated. If we assume an additional phosphorylation site is a random and independent event, the probability for any additional phosphorylation site is just the ground probability, e.g., for the S/T class, *P*_st_(O_4_ | O_3_O_2_O_1_) = *P*_st_(O_3_ | O_2_O_1_) = *P*_st_(O_2_ | O_1_) = *P*(S/T) =  0.331. Hence, the expected probability to have an additional phosphorylation site is 0.331 for the S/T class and 0.242 for the Y class. Since the first phosphorylation site is always given, the observed conditional probability for the second site is the same as the expected one. However, the observed conditional probabilities for the third and fourth site in the S/T class are *P*_st_(O_4_ | O_3_O_2_O_1_)′ = 0.554 and *P*_st_(O_3_ | O_2_O_1_)′ = 0.421, both of which are significantly higher than 0.331. This means that if the second site is phosphorylated in the same orthologous group, the third and fourth sites are more likely to be phosphorylated than random. The same is true for the Y class. The observed conditional probabilities are: *P*_y_(O_4_ | O_3_O_2_O_1_)′ = 0.375 and *P*_y_(O_3_ | O_2_O_1_)′ = 0.688, both of which are significantly higher than 0.242. A difference is that for the Y class, the peak observation is the third site when two phosphorylated sites are observed while the peak observation is the fourth site for the S/T class.

The distribution of 4T, 1S3T (one serine and three threonine aligned in the center and so on), 2S2T, 3S1T, and 4S is shown in Table 3. In the non-phosphorylated class (none of the four center aligned amino acids were phosphorylated), the distribution was 32.33, 7.19, 4.88, 10.22, and 45.38%, while the distribution in the fully conserved phosphorylated class (all of the centers were phosphorylated) is 8.82, 3.92, 2.94, 6.86, and 77.45%, respectively. In particular, only nine groups have four threonine (4T), while 79 groups have 4S in the 102 fully conserved phosphorylation S/T groups. This is consistent with the known trend that phosphorylated S is much more frequent than phosphorylated T.

**Table 3:** **Distribution of S and T in non-phosphorylated and conserved phosphorylated classes**.

	Non-phosphorylated sites		Conserved phosphorylated sites	
	Counts	Frequency (%)	Counts	Frequency (%)
4T	6,639	32.33	9	8.82
1S3T	1,477	7.19	4	3.92
2S2T	1,002	4.88	3	2.94
3S1T	2,099	10.22	7	6.86
4S	9,318	45.38	79	77.45

The table shows number of orthologous groups having at least one center predicted to be phosphorylated, the percentage of each state (1–4 predicted phosphorylation sites at the center), the expected number of groups assuming a random distribution of phosphorylation sites at the center, and the conditional probability of having an additional phosphorylation site.

**Figure 5:**
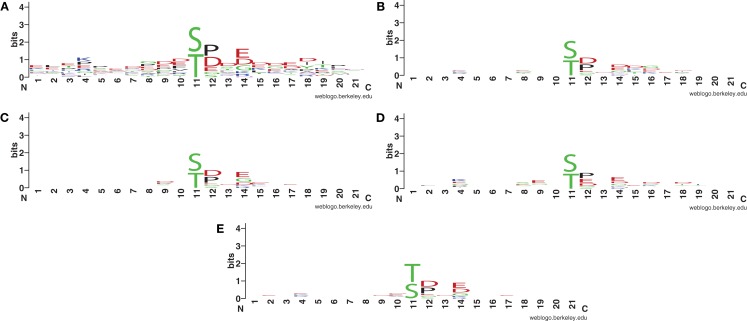
**WebLogo for S/T centered, conserved phosphorylation sites without gap in the sequence alignment: (A) overall plot for all S/T aligned sequences without gaps, (B) *A. thaliana*, (C) *O*. *sativa*, (D) *R*. *communis*, and (E) *P. patens***.

An interesting pattern to explore is the sequence profile of fully conserved phosphorylated classes. To reduce the impact of alignment gaps, some of which could be due to alignment errors by MUSCLE, we only selected those without gaps in the flanking regions to build sequence logs. The overall WebLogo (Crooks et al., 2004) plot (Figure 5A) and the WebLogo plot for each species (Figures 5B–E) are shown. In this study, a flanking sequence has a length of 21 or 10 amino acids at each side. Some over-represented amino acid types at particular locations are revealed as interesting sequence motifs. It is shown that around the phosphorylation site, more amino acids are negatively charged or polar. Amino acid Aspartate (D), Glutamate (E), and Proline (P) appear in high frequencies toward the C-terminus of the phosphorylation sites.

**Figure 6:**
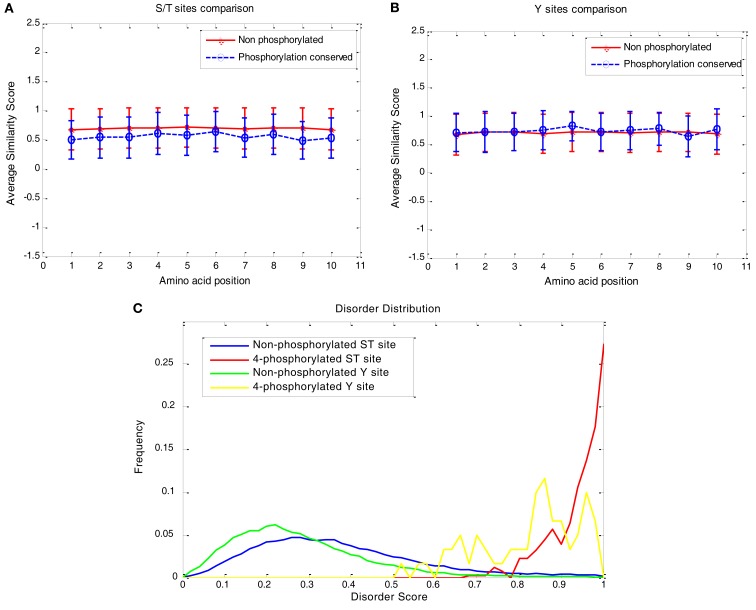
**Sequence properties at flanking regions of phosphorylation sites**. **(A,B)** Similarity score of amino acids on both sides (five amino acids each) of the flanking sequence, where the bars indicate standard deviations. It was measured by averaging the number of pairs of the same amino acid in a certain position in the alignment of an orthologous group. **(A)** For phosphorylated S/T in the center, and **(B)** for phosphorylated Y in the center. **(C)** The disorder score distribution of S/T and Y sites for exclusively non-phosphorylated and fully conserved sites.

For the S/T and Y classes, 102 and 15 groups have fully conserved centers (with all the sites predicted to be phosphorylated), respectively, and 20,535 and 6,805 groups have exclusively non-phosphorylated centers, respectively. To evaluate the sequence conservation for each class, a similarity score was calculated based on the pair-wise comparison, i.e., the pair with the same amino acid at a location of the alignment was counted as one and different amino acids as zero. The frequency of the same amino acid occurred at a position was normalized by the total number of pairs and the total number of the orthologous groups in the S/T or Y class. The mean and variance were calculated and plotted in Figures 6A,B. Surprisingly, there is no significant difference in sequence conservation between the exclusively non-phosphorylated sites and the fully conserved phosphorylation sites. The average similarity scores for the non-phosphorylated groups are almost flat at all positions for both S/T and Y class, while the scores for the fully phosphorylated group are fluctuating among different positions. The fluctuation may be due to smaller sample size for the fully phosphorylated group. In contrast, the distribution of the disorder score on the non-phosphorylated sites and the fully conserved phosphorylated sites is shown to have a major difference (Figure 6C). It is interesting to note that the distribution of the disorder score for S/T non-phosphorylated sites in the orthologous groups is similar to a right-side skewed normal distribution, while the distribution for fully conserved phosphorylation sites tends to bean exponential distribution.

## Discussion

In this work, we extended Musite for general plant phosphorylation site predictions. We tested our models extensively using six plant species. The cross-species test shows that Musite has the capability of extending the model from *Arabidopsis* to other green plants. In terms of the overall plant model, through comparison of ROC curves, the Musite predictions on both S/T and Y sites outperformed other plant-specific tools like Plantphos and PhosphAt. This comparison was conservative in that none of the testing data was included in the training set for Musite, while other tools may have included such data.

Analysis on the disorder score distributions in this study revealed different properties of serine, threonine, and tyrosine. The analysis suggests that the phosphorylation capability among serine, threonine, and tyrosine was mainly due to their relative populations in disordered regions (Figure 4A). In particular, the fact that tyrosine phosphorylation appeared less frequently than serine and threonine probably results from tyrosine’s large population in the ordered region, where the phosphorylation is not likely to occur. The observation that serine phosphorylation is much more frequent than threonine phosphorylation (Table 3) is also likely due to serine’s higher probability in the disordered regions (Figure 4A). It is interesting to note that the disorder distribution in the observed or predicted phosphosites tends to be converged, especially between serine phosphorylation and threonine phosphorylation (Figure 4B). This suggests that the same enzyme for both serine and threonine phosphorylation may require similar protein disorder in the flanking regions of the phosphorylation sites. Although the distribution gaps between the known sites and the predicted sites (Figure 4B) reveal our model’s preference for highly disordered regions, the relative trend of each amino acid is still consistent between known and predicted sites.

Analysis of predicted phosphorylation sites reveals some potential conservation patterns in phosphorylation. For example, an analysis of 1–4 phosphorylated centers using conditional probability in Table 1 indicates that the phosphorylation in the same orthologous group is not a random process. There are a large number of single phosphorylated centers. However, the probability of a third or fourth additional phosphorylation site increases dramatically. Such a bipolar distribution of isolated phosphorylation sites and highly conserved ones may indicate the evolutionary features of phosphorylation sites. The isolated phosphorylation sites may be non-functional (Landry et al., 2009) or function through fine-tuning protein’s bulk electrostatic charge in a non-positionally conserved manner (Tan et al., 2010). The prediction of Musite provides a way to study the evolutionary effect beyond the limitation of the experimental data. Although it might introduce some artifacts, the statistical analysis of the predicted phosphorylation sites may provide some valuable hypotheses, since the sample size is large and the stringent threshold with 95% specificity was used for the prediction.

Sequence similarity patterns for the fully conserved phosphorylation groups and non-phosphorylation groups show that the two groups do not have significant differences in terms of sequence conservation. In contrast, the two groups have dramatically different disorder score profiles. This suggests that phosphorylation may be driven by protein disorder much more than sequence similarity in the flanking regions. As long as the disorder property is maintained, the phosphorylation is likely to maintain during evolution. The sequence conservation pattern in the disordered region is generally weak. That may be a major reason why the sequence similarity is insignificant in fully conserved phosphorylation groups. Although our computational models introduce some bias in the disorder distribution from the known sites, it is reasonable to assume that such a bias does not invalidate the sequence conservation pattern, since the relative characteristics between the predicted and observed phosphorylation sites are probably preserved as described earlier.

Finally, we have to point out that although the computational approach provides a possibility to study the global phosphorylation pattern and trend from a large population, it does have some limitations. If a feature is weighted more than the others in the machine-learning process, then in the analysis part, this feature may dominate the pattern as a bias. For example, the dominant disorder score pattern could be due to the high weight of the disorder score feature in the predictor. Although we maintain a relatively low false positive rate, the incorrectly classified sites may introduce significant noises, or naively simplify the feature space. These may limit the capability of model’s generalization and data coverage. Separation of computational artifacts and *bona fide* biological effects requires more investigations after the machine-learning approach. Hence, while our study provides some interesting ideas and hypotheses, they call for experimental validations to further study these issues.

## Conflict of Interest Statement

The authors declare that the research was conducted in the absence of any commercial or financial relationships that could be construed as a potential conflict of interest.

## References

[B1] Benschop J. J. Mohammed S. O’Flaherty M. Heck A. J. Slijper M. Menke F. L. (2007). Quantitative phosphoproteomics of early elicitor signaling in Arabidopsis. Mol. Cell Proteomics 6, 1198–1214 10.1074/mcp.M600429-MCP200 17317660

[B2] Bi Y. D. Wang H. X. Lu T. C. Li X. H. Shen Z. Chen Y. B. Wang B. C. (2011). Large-scale analysis of phosphorylated proteins in maize leaf. Planta 233, 383–392 10.1007/s00425-010-1291-x 21053013

[B3] Blom N. Gammeltoft S. Brunak S. (1999). Sequence and structure-based prediction of eukaryotic protein phosphorylation sites. J. Mol. Biol. 294, 1351–1362 10.1006/jmbi.1999.3310 10600390

[B4] Boekhorst J. Van Breukelen B. Heck A. Jr. Snel B. (2008). Comparative phosphoproteomics reveals evolutionary and functional conservation of phosphorylation across eukaryotes. Genome Biol. 9, R144 10.1186/gb-2008-9-10-r144 18828897 PMC2760871

[B5] Chen F. Mackey A. J. Vermunt J. K. Roos D. S. (2007). Assessing performance of orthology detection strategies applied to eukaryotic genomes. PLoS ONE 2, e383 10.1371/journal.pone.0000383 17440619 PMC1849888

[B6] Chen Y. Hoehenwarter W. Weckwerth W. (2010). Comparative analysis of phytohormone-responsive phosphoproteins in Arabidopsis thaliana using TiO2-phosphopeptide enrichment and mass accuracy precursor alignment. Plant J. 63, 1–17 10.1111/j.1365-313X.2010.04261.x 20374526

[B7] Crooks G. E. Hon G. Chandonia J. M. Brenner S. E. (2004). WebLogo: a sequence logo generator. Genome Res. 14, 1188–1190 10.1101/gr.849004 15173120 PMC419797

[B8] de la Fuente van Bentem S. Anrather D. Dohnal I. Roitinger E. Csaszar E. Joore J. Buijnink J. Carreri A. Forzani C. Lorkovic Z. J. Barta A. Lecourieux D. Verhounig A. Jonak C. Hirt H. (2008). Site-specific phosphorylation profiling of Arabidopsis proteins by mass spectrometry and peptide chip analysis. J. Proteome Res. 7, 2458–2470 10.1021/pr8000173 18433157

[B9] de la Fuente van Bentem S. Anrather D. Roitinger E. Djamei A. Hufnagl T. Barta A. Csaszar E. Dohnal I. Lecourieux D. Hirt H. (2006). Phosphoproteomics reveals extensive in vivo phosphorylation of Arabidopsis proteins involved in RNA metabolism. Nucleic Acids Res. 34, 3267–3278 10.1093/nar/gkl429 16807317 PMC1904105

[B10] Edgar R. C. (2004). MUSCLE: multiple sequence alignment with high accuracy and high throughput. Nucleic Acids Res. 32, 1792–1797 10.1093/nar/gkh180 15034147 PMC390337

[B11] Engelsberger W. R. Schulze W. X. (2012). Nitrate and ammonium lead to distinct global dynamic phosphorylation patterns when resupplied to nitrogen-starved Arabidopsis seedlings. Plant J. 69, 978–995 10.1111/j.1365-313X.2011.04848.x 22060019 PMC3380553

[B12] Farriol-Mathis N. Garavelli J. S. Boeckmann B. Duvaud S. Gasteiger E. Gateau A. Veuthey A. L. Bairoch A. (2004). Annotation of post-translational modifications in the Swiss-Prot knowledge base. Proteomics 4, 1537–1550 10.1002/pmic.200300764 15174124

[B13] Gao J. Agrawal G. K. Thelen J. J. Xu D. (2009). P3DB: a plant protein phosphorylation database. Nucleic Acids Res. 37, D960–D962 10.1093/nar/gkn733 18931372 PMC2686431

[B14] Gao J. Thelen J. J. Dunker A. K. Xu D. (2010). Musite, a tool for global prediction of general and kinase-specific phosphorylation sites. Mol. Cell Proteomics 9, 2586–2600 10.1074/mcp.M110.001388 20702892 PMC3101956

[B15] Goodstein D. M. Shu S. Howson R. Neupane R. Hayes R. D. Fazo J. Mitros T. Dirks W. Hellsten U. Putnam N. Rokhsar D. S. (2012). Phytozome: a comparative platform for green plant genomics. Nucleic Acids Res. 40, D1178–D1186 10.1093/nar/gkr944 22110026 PMC3245001

[B16] Grimsrud P. A. Den Os D. Wenger C. D. Swaney D. L. Schwartz D. Sussman M. R. Ane J. M. Coon J. J. (2010). Large-scale phosphoprotein analysis in Medicago truncatula roots provides insight into in vivo kinase activity in legumes. Plant Physiol. 152, 19–28 10.1104/pp.109.149625 19923235 PMC2799343

[B17] Heazlewood J. L. Durek P. Hummel J. Selbig J. Weckwerth W. Walther D. Schulze W. X. (2008). PhosPhAt: a database of phosphorylation sites in Arabidopsis thaliana and a plant-specific phosphorylation site predictor. Nucleic Acids Res. 36, D1015–D1021 10.1093/nar/gkm812 17984086 PMC2238998

[B18] Hsu J. L. Wang L. Y. Wang S. Y. Lin C. H. Ho K. C. Shi F. K. Chang I. F. (2009). Functional phosphoproteomic profiling of phosphorylation sites in membrane fractions of salt-stressed Arabidopsis thaliana. Proteome Sci. 7, 42 10.1186/1477-5956-7-42 19900291 PMC2778640

[B19] Iakoucheva L. M. Radivojac P. Brown C. J. O’Connor T. R. Sikes J. G. Obradovic Z. Dunker A. K. (2004). The importance of intrinsic disorder for protein phosphorylation. Nucleic Acids Res. 32, 1037–1049 10.1093/nar/gkh253 14960716 PMC373391

[B20] Ito J. Taylor N. L. Castleden I. Weckwerth W. Millar A. H. Heazlewood J. L. (2009). A survey of the Arabidopsis thaliana mitochondrial phosphoproteome. Proteomics 9, 4229–4240 10.1002/pmic.200900064 19688752

[B21] Joachims T. (2002). Learning to Classify Text Using Support Vector Machines. Boston: Kluwer Academic Publishers

[B22] Jones A. M. Maclean D. Studholme D. J. Serna-Sanz A. Andreasson E. Rathjen J. P. Peck S. C. (2009). Phosphoproteomic analysis of nuclei-enriched fractions from Arabidopsis thaliana. J. Proteomics 72, 439–451 10.1016/j.jprot.2009.02.004 19245862

[B23] Kline K. G. Barrett-Wilt G. A. Sussman M. R. (2010). In planta changes in protein phosphorylation induced by the plant hormone abscisic acid. Proc. Natl. Acad. Sci. U.S.A. 107, 15986–15991 10.1073/pnas.1007879107 20733066 PMC2936636

[B24] Landry C. R. Levy E. D. Michnick S. W. (2009). Weak functional constraints on phosphoproteomes. Trends Genet. 25, 193–197 10.1016/j.tig.2009.03.003 19349092

[B25] Lee T. Y. Bretana N. A. Lu C. T. (2011). PlantPhos: using maximal dependence decomposition to identify plant phosphorylation sites with substrate site specificity. BMC Bioinformatics 12, 261 10.1186/1471-2105-12-261 21703007 PMC3228547

[B26] Li H. Wong W. S. Zhu L. Guo H. W. Ecker J. Li N. (2009). Phosphoproteomic analysis of ethylene-regulated protein phosphorylation in etiolated seedlings of Arabidopsis mutant ein2 using two-dimensional separations coupled with a hybrid quadrupole time-of-flight mass spectrometer. Proteomics 9, 1646–1661 10.1002/pmic.200800564 19253305

[B27] Li L. Stoeckert C. J. Jr. Roos D. S. (2003). OrthoMCL: identification of ortholog groups for eukaryotic genomes. Genome Res. 13, 2178–2189 10.1101/gr.1224503 12952885 PMC403725

[B28] Li W. Godzik A. (2006). CD-HIT: a fast program for clustering and comparing large sets of protein or nucleotide sequences. Bioinformatics 22, 1658–1659 10.1093/bioinformatics/btl329 16731699

[B29] Maathuis F. J. (2008). Conservation of protein phosphorylation sites within gene families and across species. Plant Signal. Behav. 3, 1011–1013 19704437 10.4161/psb.6721 PMC2633760

[B30] Meyer L. J. Gao J. Xu D. Thelen J. J. (2012). Phosphoproteomic analysis of seed maturation in Arabidopsis, rapeseed, and soybean. Plant Physiol. 159, 517–528 10.1104/pp.111.191700 22440515 PMC3375983

[B31] Nakagami H. Sugiyama N. Mochida K. Daudi A. Yoshida Y. Toyoda T. Tomita M. Ishihama Y. Shirasu K. (2010). Large-scale comparative phosphoproteomics identifies conserved phosphorylation sites in plants. Plant Physiol. 153, 1161–1174 10.1104/pp.110.157347 20466843 PMC2899915

[B32] Nuhse T. S. Bottrill A. R. Jones A. M. Peck S. C. (2007). Quantitative phosphoproteomic analysis of plasma membrane proteins reveals regulatory mechanisms of plant innate immune responses. Plant J. 51, 931–940 10.1111/j.1365-313X.2007.03192.x 17651370 PMC2156193

[B33] Nuhse T. S. Stensballe A. Jensen O. N. Peck S. C. (2004). Phosphoproteomics of the Arabidopsis plasma membrane and a new phosphorylation site database. Plant Cell 16, 2394–2405 10.1105/tpc.104.023150 15308754 PMC520941

[B34] Obradovic Z. Peng K. Vucetic S. Radivojac P. Dunker A. K. (2005). Exploiting heterogeneous sequence properties improves prediction of protein disorder. Proteins 61(Suppl. 7), 176–182 10.1002/prot.20735 16187360

[B35] Reiland S. Messerli G. Baerenfaller K. Gerrits B. Endler A. Grossmann J. Gruissem W. Baginsky S. (2009). Large-scale Arabidopsis phosphoproteome profiling reveals novel chloroplast kinase substrates and phosphorylation networks. Plant Physiol. 150, 889–903 10.1104/pp.109.138677 19376835 PMC2689975

[B36] Schwartz D. Chou M. F. Church G. M. (2009). Predicting protein post-translational modifications using meta-analysis of proteome scale data sets. Mol. Cell Proteomics 8, 365–379 18974045 10.1074/mcp.M800332-MCP200 PMC2634583

[B37] Sugiyama N. Nakagami H. Mochida K. Daudi A. Tomita M. Shirasu K. Ishihama Y. (2008). Large-scale phosphorylation mapping reveals the extent of tyrosine phosphorylation in Arabidopsis. Mol. Syst. Biol. 4, 193 10.1038/msb.2008.32 18463617 PMC2424297

[B38] Tan C. S. Jorgensen C. Linding R. (2010). Roles of “junk phosphorylation” in modulating biomolecular association of phosphorylated proteins? Cell Cycle 9, 1276–1280 10.4161/cc.9.7.11066 20234177

[B39] Wang Z. Dong G. Singh S. Steen H. Li J. (2009). A simple and effective method for detecting phosphopeptides for phosphoproteomic analysis. J. Proteomics 72, 831–835 10.1016/j.jprot.2009.03.006 19341826

[B40] Whiteman S. A. Serazetdinova L. Jones A. M. Sanders D. Rathjen J. Peck S. C. Maathuis F. J. (2008). Identification of novel proteins and phosphorylation sites in a tonoplast enriched membrane fraction of Arabidopsis thaliana. Proteomics 8, 3536–3547 10.1002/pmic.200701104 18686298

[B41] Wolschin F. Weckwerth W. (2005). Combining metal oxide affinity chromatography (MOAC) and selective mass spectrometry for robust identification of in vivo protein phosphorylation sites. Plant Methods 1, 9 10.1186/1746-4811-1-9 16270910 PMC1295590

